# The DAC‐SP Early Detection Blueprint: Catalyst for Sustainable Change in a Regional Health System

**DOI:** 10.1002/alz70860_100449

**Published:** 2025-12-23

**Authors:** Elyse Perweiler, Kevin Overbeck, David J. Libon, Mitchel A. Kling, Maryann Graham, Samuel Weiner, Mary Campagnolo, Steven Santangelo

**Affiliations:** ^1^ Rowan‐Virtua School of Osteopathic Medicine, New Jersey Institute for Successful Aging, Stratford, NJ, USA; ^2^ Virtua Medical Group, Virtua Health, Marlton, NJ, USA

## Abstract

**Background:**

Virtua Health, the largest health system in southern New Jersey, serves >140,000 Medicare patients, including 70,000 primary care patients aged ≥65 years, across 38 primary care practices. Fewer than 25% of these patients are screened annually for cognitive impairment, with only 2.5% having a confirmed diagnosis of Alzheimer's or related dementias, far below the national average of 10.9%. To address this gap, Virtua Health, through the Davos Alzheimer's Collaborative Healthcare System Preparedness (DAC‐SP) U.S. Early Detection Fellowship program, is implementing an early detection program for cognitive impairment in primary care, building on the foundational elements of its participation in the DHHS‐HRSA funded Geriatric Workforce Enhancement Program (GWEP), the CMS GUIDE model, and creating a geriatric service line.

**Method:**

The DAC‐SP Early Detection Blueprint will accelerate early detection of cognitive impairment in primary care by developing workflows and standing order sets in 5 pilot practices with plans for future expansion. Rapid Cycle Quality Improvement will address barriers, incorporate stakeholder feedback, and guide structure and process adjustments for early detection of cognitive impairment. Digitized iPad‐based cognitive screening will be integrated into Annual Wellness Visits. Positive cognitive screens will trigger clinical pathways and standing orders to assist providers in diagnosing and managing cognitive impairment. Scheduling accommodations will support providers in the early detection and diagnostic process.

**Result:**

Virtua Health hopes to increase screening of eligible patients for cognitive impairment by 30‐50% across 5 pilot practices and exceed the 10.9% national average dementia diagnosis rate. Project participation will create new care pathways and improve patient access to team‐based comprehensive dementia evaluation, advanced diagnostic modalities, care navigators, brain health programs, and disease‐modifying therapies.

**Conclusion:**

The DAC‐SP U.S. Early Detection Fellowship is a catalyst for system‐wide change, creating a sustainable model to enhance dementia care, reduce disease burden, and improve quality of life for older individuals, their families, and caregivers. Standardized care pathways, AI tools, clinician training, and stakeholder collaboration enable Virtua Health and its primary care providers to play a pivotal role in early detection of dementia, leading to improved access to disease‐modifying therapies, promotion of brain health, and better management of cognitive impairment.

